# Correction: Recent advances in MOF-based single-atom photocatalysts for CO_2_ to solar fuel conversion under sunlight irradiation

**DOI:** 10.1039/d6sc90091g

**Published:** 2026-04-17

**Authors:** Adnan Majeed, Minh-Khoa Duong, Van-Duc Nguyen, Trong-On Do

**Affiliations:** a Department of Chemical Engineering, Laval University 1065 Avenue de la Médecine Quebec QC G1V 0A6 Canada Trong-On.Do@gch.ulaval.ca

## Abstract

Correction for ‘Recent advances in MOF-based single-atom photocatalysts for CO_2_ to solar fuel conversion under sunlight irradiation’ by Adnan Majeed *et al.*, *Chem. Sci.*, 2026, https://doi.org/10.1039/d6sc00691d.

The authors regret that due to an oversight, one of the four author biographies was not included in the final manuscript. The biography text and image for Trong-On Do is shown herein.

The Royal Society of Chemistry apologises for these errors and any consequent inconvenience to authors and readers.

